# The methodology of population surveys of headache prevalence, burden and cost: Principles and recommendations from the Global Campaign against Headache

**DOI:** 10.1186/1129-2377-15-5

**Published:** 2014-01-27

**Authors:** Lars Jacob Stovner, Mohammed Al Jumah, Gretchen L Birbeck, Gopalakrishna Gururaj, Rigmor Jensen, Zaza Katsarava, Luiz Paulo Queiroz, Ann I Scher, Redda Tekle-Haimanot, Shuu-Jiun Wang, Timothy J Steiner

**Affiliations:** 1 Norwegian National Headache Centre, Norwegian University of Science and Technology, and St. Olavs University Hospital, Trondheim, Norway; 2 King Abdullah International Medical Research Center, King Saud Ben Abdulaziz University for Health Sciences, National Guard Health Affairs, Riyadh, Saudi Arabia; 3 Prince Mohammed Ben Abdulaziz Hospital, Ministry of Health, Riyadh, Saudi Arabia; 4 Department of Neurology, University of Rochester, Rochester, NY, USA; 5 Chikankata Hospital, Mazabuka, Zambia; 6 Department of Epidemiology, Centre for Public Health, National Institute of Mental Health and Neuro Sciences, Bangalore, India; 7 Danish Headache Centre, Glostrup Hospital, University of Copenhagen, Copenhagen, Denmark; 8 Evangelical Hospital, Unna, Germany; 9 University of Duisburg-Essen, Essen, Germany; 10 Department of Neurology, University Hospital, Federal University of Santa Catarina, Florianopolis, Brazil; 11 Uniformed Services University, Bethesda, MD, USA; 12 School of Medicine, Department of Neurology, Addis Ababa University, Addis Ababa, Ethiopia; 13 The Neurological Institute, Taipei Veterans General Hospital, Taipei, Taiwan; 14 Department of Neurology, Brain Research Center and Institute of Brain Science, National Yang-Ming University of School of Medicine, Taipei, Taiwan; 15 Division of Neuroscience, Imperial College London, London, UK; 16 Department of Neuroscience, Norwegian University of Science and Technology, Trondheim, Norway

**Keywords:** Burden of headache, Epidemiology, Guidelines, Methodology, Global Campaign against Headache

## Abstract

The global burden of headache is very large, but knowledge of it is far from complete and needs still to be gathered. Published population-based studies have used variable methodology, which has influenced findings and made comparisons difficult. Among the initiatives of the Global Campaign against Headache to improve and standardize methods in use for cross-sectional studies, the most important is the production of consensus-based methodological guidelines. This report describes the development of detailed principles and recommendations. For this purpose we brought together an expert consensus group to include experience and competence in headache epidemiology and/or epidemiology in general and drawn from all six WHO world regions. The recommendations presented are for anyone, of whatever background, with interests in designing, performing, understanding or assessing studies that measure or describe the burden of headache in populations. While aimed principally at researchers whose main interests are in the field of headache, they should also be useful, at least in parts, to those who are expert in public health or epidemiology and wish to extend their interest into the field of headache disorders. Most of all, these recommendations seek to encourage collaborations between specialists in headache disorders and epidemiologists. The focus is on migraine, tension-type headache and medication-overuse headache, but they are not intended to be exclusive to these. The burdens arising from secondary headaches are, in the majority of cases, more correctly attributed to the underlying disorders. Nevertheless, the principles outlined here are relevant for epidemiological studies on secondary headaches, provided that adequate definitions can be not only given but also applied in questionnaires or other survey instruments.

## Preface

The importance of epidemiological studies for headache science is increasingly recognized. Apart from enhancing our understanding of disease origins patterns, aetiology and risk factors, thereby improving opportunities for treatment and prevention, these studies inform needs-assessment, underpin service policy and gain acceptance of headache disorders as a public-health priority [1]. The methods, and quality, of published studies are variable [2,3]. The need for better and standardized methodology, supported by guidelines for the design and performance of these studies, has been evident for some years [3,4].

These recommendations have been developed with this need in mind and are for anyone, of whatever background, with interests in designing, performing, understanding or assessing studies that measure or describe the burden of headache in populations. While aimed principally at researchers whose main interests are in the field of headache, they should also be useful, at least in parts, to those who are expert in public health or epidemiology and wish to extend their interest into the field of headache disorders. Most of all, these recommendations seek to encourage collaborations between specialists in headache disorders and epidemiologists.

The development initiative came from *Lifting The Burden* (LTB) a UK-registered charitable nongovernmental organization, which directs the Global Campaign against Headache in official relations with the World Health Organization (WHO). An expert consensus group was convened by LJS and TJS with two considerations in mind: to include experience and competence in headache epidemiology and/or epidemiology in general, and to have international and cross-cultural relevance. To the latter end, members were drawn from all six WHO world regions (see Table 1).

**Table 1:** Members of the expert consensus group

African Region	Gretchen L Birbeck (GLB) (Zambia)
	Redda Tekle-Haimanot (RTH) (Ethiopia)
Region of the Americas	Gretchen L Birbeck (GLB) (USA
	Luiz Paulo Queiroz (LPQ) (Brazil)
	Ann I Scher (AIS) (USA)
Eastern Mediterranean Region	Mohammed Al Jumah (MAJ) (Saudi Arabia)
European Region	Rigmor Jensen (RJ) (Denmark)
	Zaza Katsarava (ZK) (Georgia and Germany)
	Lars Jacob Stovner (LJS) (Norway) (chairman)
	Timothy J Steiner (TJS) (UK and Norway)
South East Asian Region	Gopalakrishna Gururaj (GG) (India)
Western Pacific Region	Shuu-Jiun Wang (SJW) (Taiwan)
World Health Organisation	Somnath Chatterji (SC) (Switzerland)
World Health Organisation	Tarun Dua (TD) (Switzerland)

Publication of these recommendations follows a call for them [3] this supported by a review which identified multiple methodological shortcomings in published studies, many with adverse impact on quality. Because these recommendations should stand alone, some of the argument in this review is set out again here.

## Introduction

### Purpose

The main aim of these recommendations is to improve the quality of studies of headache prevalence and burden. Much money and time are committed to such studies. We believe that attention to the principles set out here, and adherence to the recommendations, will ensure that these resources are better spent. We further believe these recommendations will not only aid the planning and performance of new studies but also assist evaluation of previously published studies.

A second aim is to make studies more comparable. Several attempts have been made to summarize the results of different studies [2,5]. In this effort, it has become evident that studies are performed and reported in quite different ways, making it very difficult to interpret and summarize their results and, particularly, to compare results from studies performed in different settings and countries or at different times. Also, while the quality of studies is very variable, otherwise well-performed studies are sometimes reported in too little detail for the results to be useful in comparison with those of other studies. Hence, guidelines that will help in making studies more uniform as well as of better quality have been called for [3,4].

### Scope

The focus of this document is on adult studies. Many of the recommendations and accompanying discussions apply equally to studies of children and/or adolescents, but some recommendations will need adaptation. Some major issues – relating, for example, to sampling methods, diagnosis, description and measurement of burden, and consent – are specific for children. Although a short section is included for these age groups, these recommendations may not be adequate for them in all of these respects. At a later date, a lengthier section devoted to these issues will be created in collaboration with the necessary expertise. There are also some considerations specific to studies of the elderly, and attention is drawn to these where relevant.

In general these recommendations apply to prevalence of and burden generated by primary headache disorders, mainly migraine and tension-type headache (TTH). They are not intended to be exclusive to these, although burdens arising from secondary headaches are, in the majority of cases, more correctly attributed to the underlying disorders. An exception is medication-overuse headache (MOH), also secondary by definition but included because it arises, on present understanding, only in the context of a pre-existing and usually primary headache disorder and may be considered a sequela of this. Unquestionably, MOH contributes to public ill-health [6], and these recommendations do embrace it.

The recommendations also encompass other headaches occurring on ≥15 days/month, again because they certainly contribute to public ill-health. It is acknowledged later that these may be poorly characterized and, within a survey conducted by enquiries at single points in time, impossible to diagnose more specifically than as frequent headache.

Specific recommendations have not been formulated for other secondary headaches, because our understanding of many of these disorders is incomplete and evolving, their causes and mechanisms are often unknown and their diagnostic criteria – particularly in relation to causation – often too uncertain to be usable in epidemiological studies. Nevertheless, the *principles* outlined in this document are relevant for epidemiological studies on secondary headaches, provided that adequate definitions can be not only given but also applied in questionnaires or other survey instruments.

The methodological issues in studies on headache prevalence and burden arise mostly in three key areas. The first of these concerns the population of interest: how this is defined, and how the people who are part of it are sampled, reached, identified as cases or not and engaged in appropriate enquiry. The second concerns diagnosis, where this is material to the enquiry, and the collection and reporting of data that adequately describe and quantify the different components of headache burden. The third concerns quality control, data management, analysis and publication. Each of these is dealt with in these recommendations.

Lastly it should be stated that this document is not a textbook on epidemiology. There are many issues of a general nature better explained in such textbooks. We recommend that studies on headache epidemiology and burden are planned and performed in collaboration with an epidemiologist familiar with the local population and culture(s). With regard to the reporting of studies, the STROBE (Strengthening the Reporting of Observational Studies in Epidemiology) statements (http://www.strobe-statement.org/) [7,8] should be consulted. Here in this document are recommendations that relate specifically to studies of headache, or address issues of particular relevance to headache, although some more general discussion is included where it is needed to support these.

### The burden of headache

“Burden of disease” at a population level is the sum of the negative effects a disease has on the individuals within that population, together with any societal burden. It is a complex construct, composed of many different elements. Not all of the affected individuals actually have the disease.

Most primary headaches are episodic, giving rise directly but intermittently to symptom burden. In a minority of cases, symptoms and symptom burden persist more or less continuously, albeit variably in their intensity. Symptoms include pain, which may be accompanied by nausea, vomiting and photo- and/or phonophobia. While present, these symptoms may cause debility and prostration, and reduce functional ability. This secondary disability burden is of magnified importance because headache is most common in people between their teens and 50–60 years of age – the productive years. Its consequences include inability to work or, when work is continued, reduced effectiveness and productivity. Attached to this may be an individual financial burden from lost pay.

Given that headache attacks are unpleasant, it is hardly surprising that people who experience them frequently worry about when the next may occur. Neither is it surprising that this can provoke anxiety, and avoidance behaviour. People with migraine in particular may identify triggers, which they attempt to eliminate by lifestyle compromise. Leisure activities may be cancelled or curtailed because of headache; when many have been cancelled, social events are likely not to be planned in the first place. Social life between attacks may simply cease. These are elements of interictal burden, which may continuously affect wellbeing and be sufficient to impair quality of life.

A consequence of recurring inability to work may be a reputation for poor reliability, or inability to cope. Decreased probability of promotion follows, with failure to develop full career potential; in egregious cases, early retirement may be a forced result of persistent ill health. A consequence of lost school-time, affecting education, may be reduced career opportunities later. In both cases, the result is lower pay and impaired financial security. Over a lifetime, the cumulative burden of financial losses can be substantial.

People with headache bear much of these burdens, but not all. Burden on others, unaffected by headache themselves, arises in several ways. Failed or abdicated social roles during attacks affect partnership and parenthood. Family and friends lose the companionship they reasonably expect, but which is not given by a person shut away in a dark room. Children may not always be looked after. Partners and other family members may inherit increased shares of chores and responsibilities. They may acquire a carer burden, called to look after the person with headache. Carers, as well as the sufferer, can lose time from work.

Similarly, employers and work colleagues carry part of the burden of headache when paid work is not done. Either the employer pays for nothing, or colleagues must take on extra duties to make up.

Consultations with physicians, and investigations, occupy time. Health-care resource consumption may be paid for by people with headache, but, when direct treatment costs for a condition affecting over 10% of the population are reimbursed by a State-funded health system, the societal economic burden is substantial. Nevertheless, as a contributor to this element of burden, these costs are dwarfed by the indirect cost to national economies of absenteeism and reduced effectiveness at work [6,9].

### Headache epidemiology

An accepted definition of epidemiology is “the study of distribution and determinants of disease frequency in human populations” [10]. Epidemiological studies are often classified as descriptive (setting out the distribution of disease among different groups) or analytical (elucidating determinants, *ie*, risk factors or causes).

The importance of headache disorders to public health, and therefore as a subject of epidemiological study, is very evident from the description above of headache burden. Not only is this reflective of substantial public ill health but also, of course, it makes high demands for health care to relieve it. Descriptive epidemiological studies are the fundamental basis of needs-assessments for health-care provision, both for entire populations and for various subgroups within it whose needs may be different.

The usual focus of descriptive epidemiology in headache is prevalence: an estimate of how common a disease is, expressed as the total number of cases in a population (numerator) divided by the number of individuals in that population (denominator). Other important and related concepts in descriptive epidemiology are incidence (a measure of the risk of newly developing a condition within a specified period of time), which is expressed as a rate, remission (the probability of a case becoming a non-case, through natural history or intervention, again within a specified period of time), and duration (the period between onset and remission). Also related is mortality, but this has little relevance to primary headache disorders. Clearly, incidence, duration, remission and prevalence are tied together not only conceptually but also mathematically, the last being the steady-state consequence of the first two, often summarized by the formula P = ID, where P = (point) prevalence, I = incidence per year and D = duration in years.

Headache terminology is unfortunately inconsistent, owing to the fact that headache is a *chronic disorder with episodic manifestations*. An “active headache disorder” is essentially characterized by the occurrence of symptoms within the previous year [11]. Prevalence studies that adopt this definition of a case (*ie*, an individual who reports at least one headache episode during that time) necessarily use a timeframe of 1 year and usually report the findings as 1-year prevalence. Strictly speaking, however, this estimates the number of *current* cases (point prevalence). A different enquiry may define a case only when symptoms are actually present (“headache now”); this also estimates point prevalence, but of headache attacks, not of a headache disorder. The terms “incidence” and “remission” can, similarly, be applied either to headache attacks or to headache disorders, although in practice these terms have generally been used to refer to headache disorders, using specified time periods (*eg*, incidence rate = number with first onset of headache per 100 person-years; remission rate = % of cases of headache disorder who then have no further attacks during 1 year of follow-up). All of these terms must be used carefully to avoid confusion.

The large burden attributable to headache disorders should equally motivate analytical epidemiological studies to determine risk factors and causes of headache, some of which may be preventable. This may not be possible within the same studies that measure prevalence and burden, requiring, in addition, the collection and registration of relevant exposure data and follow-up studies. These requirements are often best fulfilled by larger epidemiological surveys unrestricted to a single disorder or set of disorders (see page 26).

These recommendations are of equal relevance to descriptive and analytical studies. But which of these is the purpose of the study must always be clear at the outset: it affects, fundamentally, the design of the study, the choice of study population, the size of the study sample and the information to be collected.

## Working process

Members of the expert consensus group were invited to participate in February, 2011. A review of the relevant literature had been performed already in connection with previous initiatives to document the prevalence of headache in Europe [5,12] and globally [2], the burden of headache [13], and methodological issues arising from their measurement [4]. A first draft of these recommendations was prepared by LJS and TJS and distributed to the members for comment in June, 2011, and both the draft and the comments received were debated in depth at a meeting of the group in Trondheim, Norway, during 1st-3rd September, 2011. The group drew also upon their recent experience from population-based studies conducted by *Lifting The Burden*[14-17]. A second draft emerged, and was circulated for further comments; a third draft, amended according to these, followed in early February, 2012; and a fourth draft, with comments assimilated by LJS and TJS, was approved by the group as a consultation draft in June, 2012.

Approval from the International Headache Society to post this draft on their website for public worldwide consultation was requested in June and given in November, 2012. The consultation took place throughout January and February, 2013, and appropriate comments and suggestions arising from it were incorporated by LJS and TJS in April, 2013. The resultant fifth draft was sent to group members for approval and, with final amendments, became this document. The entire process took nearly three years.

## Summary of principles

### Ethical issues

All studies should conform to the ethical principles of:

• Respect for autonomy;

• Non-maleficence;

• Beneficence;

• Justice.

A study is unethical when:

• It does not conform to norms and ethics of the country in which it is performed;

• It is conducted without consent;

• It utilizes resources wastefully.

A study may be unethical if:

• It does not respect issues of equity;

• It is under-resourced, so that its purpose cannot be achieved;

• It does not adhere to scientific principles.

### Study design

The study design should:

• Match the purpose of the study;

• Be explicitly justified;

• Take due account of available resources and the general conditions in the area(s) where the study will be performed;

• Be described in sufficient detail that the study can be replicated.

### The population of interest

The population of interest should:

• Be selected to match the purpose of the study, and the selection explicitly justified;

• Be described in sufficient detail that the study can be compared with others.

### Control of bias

• Biases are inevitable, but should be minimized.

• The likely causes of bias should be identified at outset, and due steps taken to manage them.

### Sample selection and avoidance of selection bias

• Sampling introduces multiple opportunities for selection bias, which should be recognized and controlled.

• Sampling aims, first and foremost, to produce a surveyable group, within the population of interest, who are representative of it for all variables that may influence the objects of measurement.

• The sample must be of sufficient size to achieve the study purpose(s), and not so large as to waste resources.

• Sampling identifies individuals to be surveyed but usually does not of itself provide a means of access to these individuals, while the means of access is an important consideration to take into account when determining the sampling method.

• The sampling method should be explicitly justified.

• The sampling method should be described in sufficient detail that the study can be replicated.

• Biases that might have arisen from the sampling procedure should be identified and discussed.

### Accessing and engaging participants

• Access is necessary to engage participants in the survey enquiry.

• In some cases, sampling of the population of interest and access to those identified as members of the sample are achieved simultaneously, in a single process.

• Incomplete access to all identified members of the sample introduces multiple opportunities for selection bias, which should be recognized and controlled.

• The access method should be explicitly justified.

• The access method should be described in sufficient detail that the study can be replicated.

• Access to potential participants does not of itself provide a means of engagement with them, but means of engagement is an important consideration to take into account when determining the means of access.

• Incomplete engagement of accessed members of the sample introduces further opportunities for selection bias, which should be recognized and controlled.

• The method of engagement should be explicitly justified.

• The method of engagement should be described in sufficient detail that the study can be replicated.

• Biases that might have arisen from incomplete access and/or engagement should be identified and discussed.

### Participation rate and non-participation

• The participation rate must be calculated.

• Low participation rate increases risk of selection bias.

• Likely causes of non-participation should be recognized at the outset, and controlled.

• A listing of the reasons for non-participation should be given, ideally in subgroups according to age and gender.

• Biases that might have arisen from non-participation should be identified and discussed.

### Method of enquiry

The method of enquiry should:

• Be suited to the purpose of the study;

• Encourage participation;

• Capture the necessary data;

• Take due account of available resources, the general conditions in the area(s) where the study will be performed and the characteristics of potential participants;

• Be tested in the specific setting of the study;

• Be adequately described.

### Case definition and diagnosis

• Headache caseness should be defined precisely, including its timeframe.

• The screening question should be neutral, unless a good reason exists for it to be otherwise, and reported *verbatim*.

• Diagnoses should be *based* on current ICHD criteria, but modifications are *always* necessary; these should be both explained and justified.

• Diagnostic instruments should be applied in the language of intended participants.

• Diagnostic instruments require validation in the language and specific setting of the study.

• Diagnoses should be made algorithmically from the recorded responses to diagnostic questions, and not by the interviewer.

• The value of diagnosing different headache types and subtypes should be critically assessed in the light of the study’s purpose.

### Pilot study

A pilot study:

• Identifies problems before full commitment of resources, and therefore:

•Supports optimum study design, logistic planning and effective conduct of the main study;

•Avoids potentially major wastage of resources;

•Is ethically desirable;

• Helps in training of research staff, especially interviewers.

### Burden estimation

Enquiries into burden should be:

• Relevant;

• Comprehensive (in relation to the purpose of the study);

• Comprehensible to the respondent;

• Amenable to analysis.

### Studies focused on economic burden of headache

Comprehensive enquiries into economic burden:

• Should include both direct and indirect costs;

• Must have reliable data sources for attributing costs;

• Must be based on population studies, not patient samples;

• Should preferably be planned and performed in collaboration with a health economist.

### Data collection and storage

Data management and storage processes should:

• Be designed to minimize error;

• Be carried out meticulously;

• Respect the privacy of participants and accord with the laws and regulations for data storage in the country.

### Reporting the study

• Poor reporting diminishes the value of otherwise good studies.

• Reference to the STROBE statement is recommended.

• Methods should be reported in sufficient detail to permit replication (which may require that they be published separately).

• Results should be reported in accordance with the study’s purpose(s).

• Discussion should show that the study objective was achieved, or explain why it was not.

## Ethical issues

Ethical issues in epidemiological studies arise in planning, conducting and reporting them.

### Principles

All studies should conform to the ethical principles of:

• Respect for autonomy;

• Non-maleficence;

• Beneficence;

• Justice.

A study is unethical when:

• It does not conform to norms and ethics of the country in which it is performed;

• It is conducted without consent;

• It utilizes resources wastefully.

A study may be unethical if:

• It does not respect issues of equity;

• It is under-resourced, so that its purpose cannot be achieved;

• It does not adhere to scientific principles.

### Commentary

The expert consensus group recognized the several ethical principles firmly and universally established in medical practice. These include respect for the autonomy of patients, non-maleficence, beneficence and justice [18], the last with particular reference to resource allocation in a context of limited resources (distributive justice). Medical professional ethical principles include veracity (truth-telling), fidelity (the keeping of promises) and confidentiality, and there are, in addition, more general approaches to medical ethics based, for example, on human rights, the needs of patients, the responsibilities of doctors, the good of society as a whole, and deserts. These are discussed in detail elsewhere, specifically in relation to sponsored and non-sponsored clinical research into headache [19,20].

Approval of a study by an appropriate ethics review board (ERB) is mandatory. Usually this should be obtained from a local ERB. Where such does not exist, approval is required from another legitimate, authoritative and competent source, such as the WHO Research Ethics Review Committee. Data protection (see page 7) must also be given due consideration [20], and may, according to the laws of the country in which the study is conducted, require additional approvals.

Consent in surveys is often implicit, and continuing: respondents renew consent each time they provide an answer to an enquiry. Formal written consent at the start of the enquiry provides only evidence of consent at that moment, and may serve little purpose for that reason (although an ERB may require it). Consent must be informed, which requires that the purpose and nature of the survey are explained to the participants’ satisfaction. Consent must be also voluntary, and participants allowed to withdraw from the study whenever they may wish.

Inducements to take part in a study that carries no risk of harm to the participant do not directly raise concerns: both parties benefit – the researcher from the subject’s participation and the subject from the inducement. However, they may raise concerns indirectly, since inducements are not of equal value to all potential subjects. Monetary inducements are more attractive to poorer people, and this does not respect the principle of equity. For the same reason, monetary inducements (and probably inducements of any sort) are likely to increase participation bias (see page 9).

When the inducement is the offer of needed health care, either free or to which the research subject would not otherwise have ready access, he or she may have little choice but to participate. Still the participant benefits, and, *in the absence of risk of harm*, even this may be considered acceptable. On the contrary, it may be argued that collecting data from needy people while offering them nothing in return is objectionable. Justification is forthcoming when the purpose of the survey is needs-assessment – to inform the development of health services, which, later, will be provided for the benefit of the population being surveyed. But there is an important question, which goes beyond the ethics of reward: to what extent is there a duty of care upon researchers when untreated and possibly serious illness is discovered by a survey? This question is sometimes raised – especially in low-income countries. These recommendations cannot give a general answer: this must be a matter for local ERBs. Two points can be noted: first, surveys are commonly made by lay interviewers, who do not themselves diagnose and have no skills to recognize illness, let alone do anything to alleviate it; second, research that may benefit a society cannot be made too onerous, or it will never be done.

Data protection, and consent relating to the use of personal data, generally require that participants are explicitly informed of each of the following:

• Where, in what form, how and by whom data relating to them will be held;

• Who will have access to them;

• The purpose(s) for which they will be used, with guarantees that they will be used for no other purpose (this implies that, if data are to be stored long-term for other purposes not yet foreseen, at least a general explanation of this intention should be given);

• How they will be destroyed once the purposes are achieved.

To reduce the possibility of misuse of personal data, the duration of data storage should be as short as possible. On the other hand, it is desirable, and regulators often require it, that original research data be stored for several years, for documentation and to enable detection of fraud in science.

### Resources are limited

Studies that waste them (whether financial resources or the willingness of subjects to take part) are unethical because of the opportunity cost: other studies will not be possible as a result. Under-resourced studies that cannot achieve their purpose are likely to be unethical because the resources are probably wasted.

Adherence to these recommendations should ensure appropriate allocation of resources to, and their effective use in, headache burden research. However, the need for efficiency in this context calls for careful deliberation about whether a particular new headache epidemiological study is required at all, and about the need for high diagnostic precision, large sample size and resource-demanding methods of data collection. In some circumstances, a new stand-alone study can be adequately replaced, with conservation of resources, by joining a larger health survey. Disadvantages of the latter are discussed later (see page 26).

Unscientific studies waste resources, and are likely to be unethical for this reason alone. Worse, they may generate misleading results.

## Methodological issues

Important methodological issues arise in each of the following:

• The design of the study;

• Identification of the population of interest;

• Control of bias;

• Sampling from the population of interest;

• The sample size;

• Means of access to and engagement with participants within the sample;

• Participation rate;

• Method of enquiry and survey instruments (questionnaires);

• Case definition and diagnosis;

• Timeframe;

• Burden estimation (which aspects, and how?);

• Data management.

### Study design

#### Principles

The study design should:

• Match the purpose of the study;

• Be explicitly justified;

• Take due account of available resources and the general conditions in the area(s) where the study will be performed;

• Be described in sufficient detail that the study can be replicated.

### Commentary

Most studies on headache prevalence and burden have a cross-sectional design, since their aims are descriptive. In such studies, prevalence and burden are assessed at the same point in time. Some headache epidemiological studies have more analytical aims: to define causes of or risk factors for headache. These more usually have a case–control or a cohort design. Case–control studies typically compare cases (those who have a disease) with controls (otherwise similar persons who do not have the disease) for prior exposure to one or more putative risk factors. In cohort studies, a group (cohort) of disease-free individuals are followed and assessed periodically to determine whether they have developed the disease of interest. Study subjects within the cohort are categorized according to whether or not they have been exposed to a suspected causal factor, and incidence rates are compared in the exposed and unexposed categories.

The different methodologies demanded by these approaches are explained in standard textbooks [21]. They are different mostly in how the study samples are selected; the principles for collecting data, engaging with participants and diagnosing headaches are similar. Therefore, while the present recommendations mostly concern cross-sectional studies, we believe they will be useful in all study designs.

### The population of interest

(sometimes referred to as the sampling frame; other terms better avoided, which may have similar meaning but are sometimes used differently or ambiguously, are “source population”, “target population” and “reference population”).

### Principles

The population of interest should:

• Be selected to match the purpose of the study, and the selection explicitly justified;

• Be described in sufficient detail that the study can be compared with others.

### Commentary

The population of interest is the population that it is wished to study, and includes every person so defined. It is invariably defined geographically, and often also by one or more additional characteristics.

In headache research, the population of interest is usually, but not always, the population of a whole country. It may also be of a region larger or smaller than a country. Sub-populations defined by additional characteristics may nonetheless be perfectly legitimate subjects of study: depending on the aim of the study, the population of interest may be restricted to a specific age group (*eg*, adults of working age, adolescents, school or pre-school children), to members of groups defined by ethnicity, culture or language, to workers in certain trades or professions or university students, or to people with another particular disease, *etc*. These recommendations remain relevant to studies of these more selected groups.

While they are easily accessed, and for this reason often studied, headache patient populations are rarely legitimate populations of interest, not just because they are highly selected but also because the criteria by which they are selected (often self-selected) are generally indeterminable. Thus, a study of such populations tells little about, and cannot be extrapolated to, either the general population or any more broadly-defined population. An arguable exception occurs in the case of severe headache disorders (see page 25).

#### Characteristics of the population of interest

These (distributions of age, gender, educational levels and socioeconomic status, proportions living in rural and urban areas, *etc.*) cannot always be known, but it is advantageous if they are. Such knowledge allows evaluation of representativeness of the participating sample, and statistical adjustments with regard to these features when necessary (see below and page 15).

### Control of bias

#### Principles

• Biases are inevitable, but should be minimized.

• The likely causes of bias should be identified at outset, and due steps taken to manage them.

### Commentary

Bias refers to systematic rather than random error. It is somewhat unavoidable, but not necessarily of consequence: this depends on its magnitude and on whether it is differential (affecting some participants more than others) or non-differential (affecting all participants equally). The potential for bias, and the need to avoid or at least minimize it, drive many aspects of the planning, execution and analysis of a study.

Biases in epidemiological studies are of two main types: selection bias and information (or measurement) bias.

Selection bias, or a systematic error in taking the sample, is commonly the result of an imperfect sampling procedure and/or a low participation rate. The result may be a sample that is unrepresentative of the population of interest. With regard to gender and age composition, statistical adjustments can be made if these properties are known for the population of interest (see above and page 15 and 28). However, people suffering from headache are more likely to participate in headache studies because they have more personal interest in them. This can give rise to a form of selection bias usually termed *participation bias* or *interest-related bias*.

Sometimes the extent of selection bias can be estimated in a study among non-participants (see page 15).

Information bias, or a systematic error in measuring disease or exposure status, may be introduced when the way in which information is gathered varies systematically: for example, when two interviewers, one in a city and the other in a rural area, do not perform the interview in the same way, consequent differences in results may be erroneously attributed to area of habitation. Similarly, if the same interviewer does all interviews in one location first, and then all those in another, systematic differences in the manner of interviewing over time (*eg*, due to a learning curve) may again yield spuriously different results in the two locations.

### Sample selection and avoidance of selection bias

#### Principles

• Sampling introduces multiple opportunities for selection bias, which should be recognized and controlled.

• Sampling aims, first and foremost, to produce a surveyable group, within the population of interest, who are representative of it for all variables that may influence the objects of measurement.

• The sample must be of sufficient size to achieve the study purpose(s), and not so large as to waste resources.

• Sampling identifies individuals to be surveyed but usually does not of itself provide a means of access to these individuals, while the means of access is an important consideration to take into account when determining the sampling method.

• The sampling method should be explicitly justified.

• The sampling method should be described in sufficient detail that the study can be replicated.

• Biases that might have arisen from the sampling procedure should be identified and discussed.

### Commentary

In an ideal study, everyone in the population of interest would be included. Occasionally this has been done [22], but in most cases it is not a realistic possibility. Instead it is necessary to choose from the population of interest a smaller, manageable group of people (the sample) to whom access is possible. The essential requirement of this sample is that it should remain representative of the population of interest, because the intention is to generalize the data from it to the whole population of interest. “Representative” means similar to the population of interest in all properties of relevance to (*ie*, likely to influence) the objects of measurement (here, headache prevalence and/or burden). There is an assumption here that knowledge exists of what these properties are, which may not be entirely true. In the context of headache, representativeness clearly encompasses age and gender, which are known to affect headache prevalence, probably should encompass socioeconomic class, employment status, area of habitation (rural or urban) and ethnicity, and possibly, in some settings, should also encompass native language and/or tribal group. Methods that ensure or at least optimize representativeness with regard to a range of identified variables such as these are likely, although not certain, to achieve the same with other, unrecognized variables.

How to obtain a sample representative of the population of interest is a general issue for all epidemiological studies, which is dealt with in standard textbooks [21]. The main principles and common sampling strategies are considered here in the context of headache.

#### Probability sampling

In probability sampling methods, each member of the population has an initial probability (which is larger than zero) of being selected, and this probability can be accurately determined. These methods include random sampling, systematic sampling (every n^th^ person in a random list), stratified sampling and (multistage) cluster sampling (see page 10). All depend on random selection, and allow calculations of sampling error and valid inferences from the sample about the population from which it was drawn. The penalty is that, in general, they are more resource-consuming than non-probability sampling methods.

In simple random sampling, all individuals within the population of interest have an equal probability of being selected. The method is vulnerable to sampling error: by chance, related inversely to sample size, important characteristics of the sample such as gender or age distributions may not well reflect those of the whole population. Stratified sampling reduces this chance by dividing the whole population into sub-populations (strata), different with regard for example to age, gender and/or habitation, and randomly drawing the sample from within each of these strata, in parts in proportion to their size.

Both random and stratified sampling are relatively easy when an overview of the population of interest exists. An overview is usually in the form of a register of all members of it. Selection can then be made directly from the register (usually by computer). In some countries, a census, repeated periodically, lists all inhabitants (except illegal immigrants, who may or may not be of interest within the study). Other registers that encompass most of the population of a country or area may be electoral rolls, telephone directories, registers of health insurance companies and lists drawn up by commercial market-survey or polling companies. When the workforce of a company is the population of interest, the list of employees is such a register. For stratified sampling, a useful register must include not only identities but also, for each listed person, the characteristics upon which stratification is to be based (*eg*, age, gender, habitation).

An overview of the population need not take the form of a list of names: a map showing all households in an area to be sampled can serve the same purpose. Sometimes a new map of residences must be made, or old maps of an area updated, before a valid selection of residences is possible.

Sampling by telephone is an established method [23]. Where telephones (landline or mobile) are widespread, but no population overview exists in the form of a complete telephone directory, dialling area code(s) followed randomly by as many digits as are typical for phone numbers in the area(s) (random digit-dialling) is an effective method of obtaining a random sample [23]. In many settings, however, this method risks bias because telephones are not evenly distributed among different age, gender and socioeconomic groups. It is important for the calculation of participation rate (see page 14) to register which numbers dialled turn out to be real telephone numbers and which are non-existent.

Cluster sampling is an alternative to these methods. It is useful in areas with no pre-existing overview of the population, but often preferable anyway because it is logistically efficient, reducing travel costs and time for the interviewer when access methodology requires that participants are visited. Usually, cluster sampling involves selecting participants only from a limited number of defined geographical areas (*eg*, blocks, streets or parts of villages, or perhaps schools) that are themselves chosen randomly. Areas can also be stratified according to socioeconomic status, urban/rural location, *etc*. In multistage cluster sampling, smaller areas are selected randomly within larger areas, and this is repeated in many stages until the requisite number of small, surveyable areas are identified, spread around the region or country. In these ultimate units, all individuals, or a random selection of individuals or households, are contacted.

Cluster sampling often makes use of maps of residential areas in order to select the sample.

#### Contacting households rather than individuals

A household contains a group of people, often but not always a family, living together (defined as sharing a common kitchen). Special issues arise when sampling employs and is dependent upon methods of access (see page 12) that lead to contacts initially with households rather than single persons (*eg*, calling door-to-door or by household telephone without prior warning). Generally, in headache studies, only one of a family should be selected, because members of families are similar genetically, share their environment and have common lifestyles, effectively reducing variance when two or more are included. (Of course, this is not the case when the intention is to study familial occurrence). Selection bias can arise very easily, because certain types of person within a household are more likely to stay at home, open the door or answer the phone. This may vary according to time of day. Consequently, rather than select whoever happens to answer, the interviewer should list all members of the household, determine who among them are eligible and then select the participant randomly from those that are.

#### Replacements

The study protocol should set out the method for selecting replacements when it is impossible to contact a selected person. This can be achieved by pre-selecting more individuals than needed within each stratum, or more residences within each area, allowing for a defined non-contact rate based on expectation (perhaps informed by a pilot study [see page 21]). Otherwise it can be achieved by extending the sampling process (for example, by visiting more randomly-selected households than were initially specified).

As a prior requirement, the protocol should define “impossible to contact” in a way that avoids bias. This is dealt with below, under “Accessing and engaging participants”.

#### Non-probability sampling

With non-probability sampling methods, participants are selected from the population in some non-random manner, so that some members have reduced or no chance of being selected while others have enhanced chances, or the probability of selection cannot be determined. Methods include convenience sampling (selecting the part of the population that is conveniently to hand – patient samples being an example), judgment sampling (selecting those who are judged most likely to provide the information of interest), quota sampling (selecting quotas of the population fulfilling particular traits) and snowball sampling (allowing selected participants to recruit future participants among their acquaintances).

In non-probability sampling, selection bias is always there; the degree to which the sample differs from the population of interest is uncontrolled and may be large. It may be knowable, by comparing the sample for key characteristics (age, gender, *etc.*) with the whole population using published statistics, but, generally, selection bias is impossible to assess. Hence, if non-probability sampling is the only available option, serious consideration should be given to whether the study is worthwhile.

#### Sample size determination

Sample size depends on the prevalence of the disorder and the precision of the estimates needed, the latter being related to the purpose of the study. Additionally and importantly, sample size raises issues of resources and of ethics (see page 5). A larger sample clearly requires and consumes more resources. It is an unethical waste of resources to use unnecessarily large samples. On the other hand, it is futile, and therefore unethical, to use samples too small to support the purpose of the study.

In almost all cases, the required sample size is independent of the size of the population. An exception occurs when the population is very small, so that the sample is a large proportion of it (*eg*, the few hundred employees of a company). This is because the probability for any individual of being selected changes significantly as the sampling process continues.

As to precision, Table 2 shows estimates of the margin of error for sample sizes ranging from 10 to 10,000. The margin of error is dependent on the prevalence of the condition under scrutiny. With decreasing prevalence the absolute margin of error decreases, but the relative margin of error (*ie*, margin of error/prevalence) increases. For a condition with a prevalence of 50% it appears that, by increasing the sample size from 100 to 1,000, the margin of error is decreased from 10% to 3.2% (absolute). Thus, if the sample size is 1,000, and 50% report headache, there is 95% probability that between 46.8 and 53.2% of the total population have headache. This is acceptable precision for many purposes, while the gain from sample sizes of >2,000 is small and often not worthwhile.

**Table 2:** Margin of error* (95% **confidence interval) according to sample size and prevalence of the condition**

**Sample size (n)**	**Prevalence of condition**				
	**50%**	**30%**	**10%**	**3%**	**1%**
10	31.6	29.0	19.0	10.8	6.3
20	22.4	20.5	13.4	7.6	4.4
50	14.1	13.0	8.5	4.8	2.8
100	10.0	9.2	6.0	3.4	2.0
200	7.1	6.5	4.2	2.4	1.4
500	4.4	4.1	2.7	1.5	0.9
1,000	3.2	2.9	1.9	1.1	0.6
2,000	2.2	2.0	1.3	0.8	0.4
5,000	1.4	1.3	0.8	0.5	0.3
10,000	1.0	0.9	0.6	0.3	0.2

*calculated as 2 standard deviations (SDs) of the prevalence where SD = √(prevalence*[1-prevalence]/n).

However, if the aim is to investigate a relatively rare condition with an expected prevalence of 1%, an absolute margin of error of 0.6% may be very unsatisfactory, in which case the sample size must be increased considerably. The same is true when estimates within or comparisons between subgroups (*eg*, men *vs* women, rural *vs* urban) are part of the purpose: the sample size must be calculated to include sufficient of the population in the smallest subgroup. To avoid inflating the overall sample unnecessarily, it is possible to “oversample” persons of that particular subgroup (*ie*, select more than the proportion in the population). Persons of this particular subgroup then have a higher (but known) chance of being selected than those in other subgroups that were not oversampled, and correction is necessary when calculating overall prevalence.

A larger sample may also be needed to estimate burden than to estimate prevalence, because burden is not distributed equally among cases: most of it is accounted for by a minority of those with the disorder. Thus, 3-4% of the population have most of the burden of migraine [24]; among all people with migraine, TTH or MOH, the relatively few with MOH have the highest individual burden [6].

Cluster sampling is assumed to reduce natural variance, and therefore requires larger sample sizes to obtain the same precision of estimates [25].

### Accessing and engaging participants

#### Principles

• Access is necessary to engage participants in the survey enquiry.

• In some cases, sampling of the population of interest and access to those identified as members of the sample are achieved simultaneously, in a single process.

• Incomplete access to all identified members of the sample introduces multiple opportunities for selection bias, which should be recognized and controlled.

• The access method should be explicitly justified.

• The access method should be described in sufficient detail that the study can be replicated.

• Access to potential participants does not of itself provide a means of engagement with them, but means of engagement is an important consideration to take into account when determining the means of access.

• Incomplete engagement of accessed members of the sample introduces further opportunities for selection bias, which should be recognized and controlled.

• The method of engagement should be explicitly justified.

• The method of engagement should be described in sufficient detail that the study can be replicated.

• Biases that might have arisen from incomplete access and/or engagement should be identified and discussed.

### Commentary

Once identified, members of the sample must be contacted (access), and their willing commitment to the enquiry procured (engagement). Upon the latter depends how carefully and completely they will respond and, therefore, data quality. In many cases, procedures to identify and access participants are the same, or closely bound together. However, sampling from registers does not assist access to those subjects.

#### Access

Means of access clearly depends on what means of communication exist in the area, on availability of telephone registries, e-mail or home address lists and/or up-to-date maps of residential areas, and on infrastructure (*eg*, means and ease of travel). Well-tried and potentially effective methods include visiting door-to-door and calling by telephone (landline or mobile), both usually done without prior warning (cold-calling). Alternatively, letters may be sent by mail or e-mail when participants have been selected from registers that provide addresses. In such cases, in some settings, participants can be invited to come to the office of the interviewer. All of these are compatible with probability sampling. Other methods involve non-probability sampling: for example, stopping prospective participants in the street. This results in a convenience sample, as does using lists of telephone numbers or e-mail addresses that happen to be available rather than complete registries.

Cold-calling at households tends to yield a higher participation rate (among people who are at home) than telephone interviews, which are easier for the interviewee to terminate. In both cases, selection bias can arise easily, because certain types of person within a household are more likely to stay at home, open the door or answer the phone. Avoidance of this bias is discussed above (see page 9).

Accessing participants by mail is cheap, and by e-mail even cheaper, but these methods have two major drawbacks. First, they presume the use of self-administered questionnaires (see page 13). Second, selection bias is unavoidable because certain types of people are inherently less likely to reply.

Inviting prospective participants to the office of the interviewer is highly problematic. In favour of this approach is that participants not only are seen face-to-face but also can be examined when necessary. This may be the only practical access method when detailed interrogation, examination or investigation is required. In other circumstances it is not ideal: while convenient for the interviewer, it is time-consuming for the participant, which risks both lowering the participation rate and introducing bias with regard to who are willing and have the time.

#### Repeated attempts at access

As a means of increasing participation rate, and perhaps reducing participation bias, multiple attempts may be necessary to contact persons who do not answer first time: by sending one or more reminder letters, or by repeating telephone calls or house visits. The study protocol should define not only how many (commonly three) but also when additional contact attempts are made before a person or a household is deemed impossible to contact.

Repeated contacts have limited effect on selection bias in responses to letters or e-mails. But selection bias in cold-calling can and should be controlled, especially bias potentially arising because certain types of household are more likely to be empty, or their phones unanswered, at particular times of the day (*eg*, working households will be selectively uncontactable during normal working hours; older people may not open doors to strangers in the evening). The protocol should adopt a systematic approach to this, stipulating a schedule for repeat contacts that makes provision for these and other factors that are likely.

#### Engagement

Engagement entails the procurement of willing cooperation, so that the enquiry, which usually means obtaining responses to a questionnaire, can be completed. Success or failure in engagement directly affects participation rate and, therefore, participation bias (see page 9). Inducements to participate (see page 7) may increase *both* participation rate and participation bias.

While the method of engagement depends upon the means of access, it is also necessarily determined by certain characteristics of the prospective participants: in particular, literacy, language, culture and cognitive ability.

Administration of a questionnaire or other survey instrument can be achieved by interview, which may be face-to-face or by telephone. Self-administered questionnaires can be mailed or e-mailed, handed out in certain settings (school, workplace, doctor’s office, *etc.*) or distributed by internet. Sometimes, subjects may be engaged in groups or through a third person.

Face-to-face interviews are the most direct method of engagement, and, uniquely, allow participants to be examined. They are almost the only method useful in populations with poor literacy. Their big drawback is that they are time-consuming and therefore expensive. Telephone interviews are almost as direct but, of course, physical examination cannot be performed. Both face-to-face and telephone interviews allow clarification of questions. This is generally thought to lead to more accurate answers, but clarification can give rise to information bias, firstly because the information given to those who ask is different from that given to those who do not, and secondly because different interviewers may give different clarifications. Therefore, if clarification is allowed, there should be clear, pre-specified limits to the extent of it, aiming to ensure that all interviewers do it in the same way. The pilot study (see page 21) is a means of anticipating these potential difficulties, identifying which questions may cause trouble and the nature and extent of clarifications they may require.

In a computer-assisted telephone interview (CATI), lay interviewers follow a script driven by a computer program; the responses are entered directly into the computer and in-built branching logic will skip questions that are not relevant according to previous answers. Face-to-face interviews can be computer-assisted in the same way. Otherwise, similar methodology can be incorporated into face-to-face interviews by careful questionnaire design. These methods permit only pre-scripted clarifications.

Self-administered questionnaires are a relatively cheap method, but one that requires a high degree of literacy and some familiarity with answering questionnaires. The lack of engagement with an interviewer provides no encouragement to respond and no opportunity for clarifications; participation rates are generally low, and incomplete returns are common.

Engagement in groups can be a cost-effective way of performing a study in some settings (*eg*, by a teacher posing questions about or descriptions of headaches collectively to the pupils in a classroom). Engagement through a third person may be the only way to gain contact with or information from some participants. Among small children, most or part of the information must be obtained through parents, or from teachers in the case of school children. In some cultures, people affected by headache can be engaged only through village elders or heads of households. All such methods risk misunderstandings, and this risk must be evaluated. Additionally, lower sensitivity and specificity for detecting headache must be expected from such remote engagements than from making direct contact with everyone in the sample individually.

#### Interviewers

Careful selection and adequate training of interviewers are of paramount importance, whether interviews are conducted face-to-face or by telephone.

As to selection of interviewers, interviewing is a skill in itself, and it should not be assumed that health personnel such as nurses, medical students or, especially, doctors are the best qualified to do it. Unless the interviewer is a headache specialist (defined here as a physician skilled in headache diagnosis and familiar with the culture and language of the respondent), diagnoses should be made *not* by the interviewer but by later applying an algorithm to the questionnaire responses (see page 20). It is therefore doubtful, in most surveys, whether clinical skills are important: it may be better to engage professionally-trained interviewers who understand interview methodology, are given basic understanding of the purposes of key questions and who follow the questionnaire and operations manual.

On the other hand, if the interviewer *is* a headache specialist, multiple diagnoses can be made in the same patient, where appropriate, and, if examination and supplementary investigations are made, secondary headaches can be diagnosed. No validation of the diagnostic method is needed (see page 20) since it can be assumed that optimal diagnostic methods are employed. Headache specialists, of course, may not explicitly use ICHD diagnostic criteria: they have at their disposal and are likely to apply, as in the clinic, a broader, experienced-based and more inclusive set of criteria for diagnosis, which ICHD-based questionnaires can never match. High sensitivity can be expected for detecting relatively minor headache complaints and rare headaches.

In most low-income countries, headache specialists are simply not available.

Adequate training embraces a clear understanding of the nature and purpose of the survey, and a recognition of which questions are of particular importance or may need clarification (see page 13). If several interviewers are used, training should be identical for each of them to ensure that data are collected in the same way by all, without introduction of information bias (see page 9). While multiple interviewers almost inevitably cause some degree of variability, more interviewers reduce the duration of the study, which, if long, can also be a source of variation.

### Participation rate and non-participation

(avoided terms: response rate, responder rate).

#### Principles

• The participation rate must be calculated.

• Low participation rate increases risk of selection bias.

• Likely causes of non-participation should be recognized at the outset, and controlled.

• A listing of the reasons for non-participation should be given, ideally in subgroups according to age and gender.

• Biases that might have arisen from non-participation should be identified and discussed.

### Commentary

**Figure 1:**
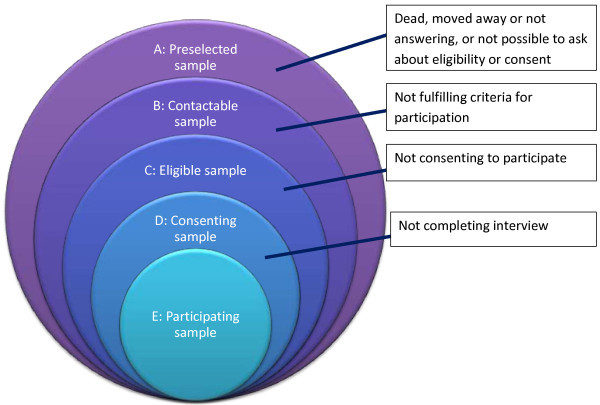
Definition of participating sample.

**Figure 2:**
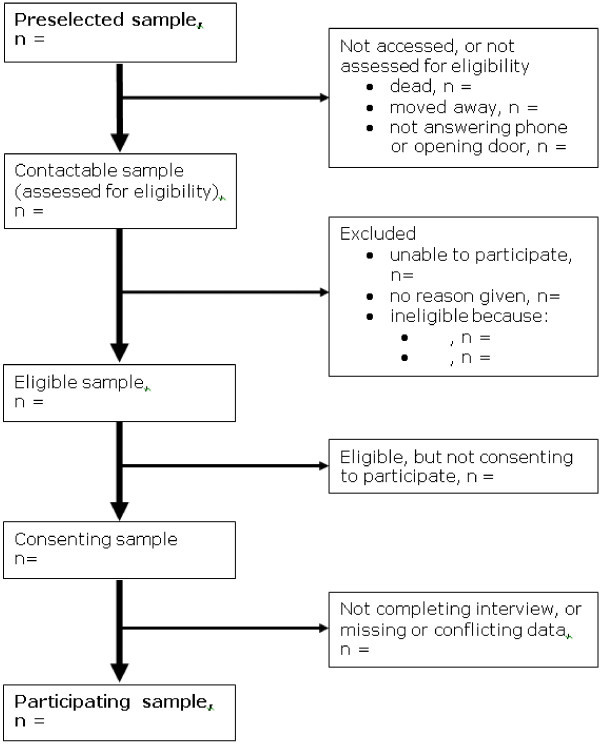
Flowchart of participation.

Although no universal definition of it exists [8], participation rate (strictly a proportion rather than a rate) is generally understood as *the proportion of those selected, contacted and eligible who actually participate meaningfully in the study*. In Figure 1, it is given by E/C. As this figure shows, calculation of the participation rate excludes those in the preselected sample who were not contactable because they had died or moved away since the study was planned, or because no-one answered the phone or opened the door. It also excludes those who were contacted but found to be ineligible because it was not possible to ask them for consent, they did not fulfil the eligibility criteria (*eg*, wrong age or gender) or it could not be determined whether or not they fulfilled the eligibility criteria. A flow diagram is an effective way to demonstrate how the participating sample was made (see Figure 2).

In almost all cases, non-participation results principally from refusal. Additionally, some of those who consent to participate may nevertheless be unable to cooperate usefully, perhaps answering only a few questions or providing conflicting responses.

#### Bias through non-participation

Only a very high participation rate *guarantees* representativeness of the participating sample. This is rarely achieved, except among “captive” samples such as pupils interviewed during school hours. Participation rates of ≥80% and ≥70% are considered excellent and acceptable respectively, but even these do not automatically secure representativeness. On the other hand, a low participation rate does not necessarily mean that the participating sample is unrepresentative: this depends on the factors responsible for non-participation [8]. Because participation rate tends to vary between different subgroups of the eligible sample (*eg*, it may be particularly low among young males), it influences the overall results (*ie*, is a source of bias). In other words, both how many and who actually participate in the study are crucial to representativeness of the participating sample.

For these reasons, participation rate needs to be known overall (E/C in Figure 1), which is relatively easily achieved. Ideally it should be known also in relevant subgroups. This requires collection of a minimal dataset from non-participants (see below), including the characteristics most likely to influence the object of measurement (age, gender and socioeconomic status, when this object is headache). This may not be possible. In default, comparisons for these characteristics between the participating sample and the population of interest can be made, if they are known for the latter. Then, within limits, imbalances can be corrected statistically by weighted adjustments of the raw data (see pages 9 and 28).

Furthermore, all reasons for non-participation (*eg*, declined to participate, too sick to be interviewed, did not return questionnaire, questionnaire not filled in appropriately, *etc.*) should be listed, and taken into account in evaluating representativeness of the participating sample and the likelihood, magnitude and influence of resulting bias.

A pilot study may establish the likely participation rate before all resources are committed to a study that may fail (see page 21). It also anticipates causes of non-participation.

#### Non-participant study

When participation rate is low, representativeness may sometimes be estimated through a study of non-participants. Such studies are never easy to perform but are possible, in some settings, when the initial sampling was from a register including contact details. Enquiry is usually by telephone, calling a random sample of non-participants. It is necessarily limited to a few key questions (*eg*, age, gender, the screening question(s) for headache [see page 18], perhaps one on headache frequency and a very few on diagnosis). This minimal dataset will at least show whether non-participants are similar to or very different from participants in the main study, and therefore is highly valuable in assessing various types of selection bias. An additional question asking why they did not participate initially will also inform discussion of selection bias, and may help improve the design of future studies.

Unfortunately, such studies are impossible among people not pre-registered, since they cannot be accessed.

### Method of enquiry

#### Principles

The method of enquiry should:

• Be suited to the purpose of the study;

• Encourage participation;

• Capture the necessary data;

• Take due account of available resources, the general conditions in the area(s) where the study will be performed and the characteristics of potential participants;

• be tested in the specific setting of the study;

• be adequately described.

### Commentary

One or more study instruments guide the process of enquiry so that it captures all of the data needed for the study’s purpose(s).

#### Study instrument

A structured questionnaire (*ie*, prescribed questions with predefined answer options) is usual, both for headache case diagnosis and for enquiry into headache burden. To some questions, open answers (*eg*, number of days with headache, or names of medicines) are preferable; otherwise, open questions may be difficult to interpret and categorize and, for diagnosis, do not permit algorithmic determination. An alternative approach to diagnosing headache is a recognition-based method, presenting descriptions (case vignettes) or pictorial representations of different headaches [26].

In a structured questionnaire, depending on the study purpose(s), any or all of the following elements may be needed:

• Identifier(s);

• Demographics: age, gender, education, employment, income (personal and/or household), habitation (urban, rural), ethnicity (when relevant), *etc.*;

• Screening and sieve questions;

• Diagnostic questions;

• Questions on headache symptom burden: headache frequency, duration, intensity, *etc.*;

• One or more other elements of burden depending on the purpose(s) of the study: *eg*, disability, time loss, family burden, cost, *etc*.

Irrelevant questions are irritating to participants, and create unnecessary workload. Questionnaires should be parsimonious, and not include any questions that do not contribute to the study’s purpose(s). Further, questionnaires should avoid enquiries that are individually irrelevant, by directing respondents past sections that are not applicable. CATI methods (see page 13) are designed expressly to do this, but it is not difficult to construct written questionnaires to do so also.

If all questions are pertinent to the survey, as they should be, each one needs to be answered clearly by each participant. However, there are certain questions such as the screening question (see page 18), diagnostic questions and key questions on burden that cannot be allowed to remain unanswered if the respondent is not to be categorized as a non-participant (see page 15). These should be highlighted to the interviewer.

Recognition-based diagnosis (presenting descriptions or pictorial representations of different headaches) is appropriate and convenient – and may give the most accurate results – in certain settings and populations (especially young children). But *mixing* recognition-based and question-based enquiries is unwise, because the former is highly leading: if an image depicting unilateral headache is recognized, it cannot be followed by the question “Is your headache on one or both sides?” [27].

#### Preparing and testing the questionnaire

The quality of the questionnaire is fundamental to the quality of the entire study: nothing can compensate for failures in data collection. Time and resources are well spent in developing a good questionnaire.

The following should be considered:

1) **Content**

How many diagnoses are necessary, and what aspects of burden are to be measured?

2) **Layout**

Clarity and ease of use reduce error rates.

3) **Intelligibility and acceptability**

The need for these, to encourage participation and capture the necessary data, is obvious; but they are not readily achieved, and it should not be assumed that they have been. A pre-pilot study is recommended to show that questions are culturally inoffensive and that the length of the questionnaire is acceptable. For this limited purpose, a small convenience sample can be drawn from patients in clinic, and, if the questionnaire has been developed in English, a headache-expert interviewer can translate questions as they are asked. A larger pilot study (see page 21) is needed after translation to test whether the questions are understood correctly and discover those that may cause problems and require clarification (see page 13). This study can also use a convenience sample, but it *must be drawn from the population of interest*. Feedback from it may show that the questionnaire needs amendment(s) (and re-translation and re-testing).

#### Translating the questionnaire

Questionnaires should almost invariably be presented in the native language of the intended participants. Many of the key concepts used in the diagnosis of headache, even the concept of headache itself, have variable meanings in different languages. Meticulous translation is necessary, and requires a rigorous translation protocol with backward and forward translations [28].

#### Validating the questionnaire

Validation (proof in use) of at least the diagnostic part of the questionnaire is highly desirable in the language and specific setting of the study. Recommendations with regard to this are detailed in the section below on case definition and diagnosis.

#### The HARDSHIP questionnaire: standardized questions for prevalence and burden studies

This modular questionnaire for studies of headache prevalence and burden, published alongside these recommendations [29], is an example of a survey instrument including the elements described above and following the principles set out in the sections below. It has been translated into several languages and used in many countries with diverse cultures. It includes a diagnostic module based originally on ICHD-II (and now on ICHD-3 beta [30], with necessary modifications (see page 18), and validated against headache experts’ diagnoses in many different settings.

### Case definition and diagnosis

#### Principles

• Headache caseness should be defined precisely, including its timeframe.

• The screening question should be neutral, unless a good reason exists for it to be otherwise, and reported *verbatim*.

• Diagnoses should be *based* on current ICHD criteria, but modifications are *always* necessary; these should be both explained and justified.

• Diagnostic instruments should be applied in the language of intended participants.

• Diagnostic instruments require validation in the language and specific setting of the study.

• Diagnoses should be made algorithmically from the recorded responses to diagnostic questions, and not by the interviewer.

• The value of diagnosing different headache types and subtypes should be critically assessed in the light of the study’s purpose.

### Commentary

In studies whose purpose is to assess headache prevalence, or describe its characteristics, case definition or caseness (*ie*, what precisely is meant by “headache”) is of obvious and fundamental importance. It can greatly influence the results. Of equal importance is clarity about how headache, and any of its types or subtypes that are relevant to the study, are diagnosed (see page 18 and page 19). For example, “migraine” in some studies has included all of its subtypes (ICHD-II codes 1.1-1.6) but in others only migraine with or without aura (ICHD-II codes 1.1 and 1.2). “Migraine with aura” itself must be clarified because, within ICHD-3 beta, it includes the subforms *migraine with typical aura*, *migraine with brainstem aura* and *typical aura without headache*. The terms “migraine” and “tension-type headache” have in some studies included both episodic and chronic forms of each and in others been restricted to the episodic forms, with the chronic forms subsumed within the general category of headache occurring on ≥15 days/month.

#### Timeframe of headache

Caseness usually implies the presence of an *active* headache disorder, which was defined in ICHD-II as characterized by any occurrence of headache (either generally or of the requisite type) during the last year [11]; hence 1-year prevalence is most used, and it allows the most comparisons with previous studies (but see page 4). Shorter timeframes (6-month and 3-month) are also quite common; while they yield data on active headache disorders, some of those characterized by low-frequency recurrence are missed (which may not matter).

The limitations of recall (correctly remembering what happened when) are an important factor in the generation of information bias. They are greater in the elderly, so introducing differential bias (see page 9). They are also, obviously, of greater impact over longer periods. Recall of headache *occurrences* during the previous 3 months is presumably more accurate than recall of those during the last year; this is certainly true of recall of the *characteristics and consequences* of each occurrence. Very short and recent timeframes such as 1-day prevalence (ascertained by questions such as “Do you have headache today?”, or “Did you have headache yesterday?”) avoid recall problems almost altogether. An estimate of 1-day prevalence does not, alone, describe the proportion of the population with an active headache disorder (for discussion of the potential terminological confusion relating to this, see page 4) but is able to yield very accurate information on diagnosis, function and other aspects of burden (see page 24).

As to headache yesterday, this is most correctly estimated when respondents are contacted directly by face-to-face interview or telephone, and not given the opportunity to choose when to answer. If the question is posed by letter, for example, it is conceivable (indeed not unlikely) that persons with headache on the day of receiving it will postpone answering until the first headache-free day, resulting in a falsely-high reported prevalence of headache yesterday.

Lifetime prevalence (“Have you ever had headache?”) includes both those with active headache and those who have previously had headache but no longer do so. As already observed, recall of headache long ago is problematic; this is true especially in the elderly while, in the young, not only may recall be better but also there is no “long ago”. Lifetime prevalence has mostly been used for, and is of most interest in, the rarer headache disorders such as cluster headache, because it broadens the definition of caseness and so increases the likelihood of observing cases. Lifetime prevalence is very relevant also in genetic epidemiological studies, but for a different reason: there is need to eliminate those who have ever had the disorder from control groups.

A single study can ask participants about each of lifetime, 1-year and 1-day prevalences.

#### Case ascertainment: screening and sieve questions

Most studies use a two-stage procedure: participants are first asked whether they have headache or not (screening question), and only those answering affirmatively are posed the more detailed diagnostic questions. This is time saving, and avoids irrelevant enquiry (see page 16), but there is a potential penalty: a negative answer to the screening question terminates further enquiry even though it can be false. In some studies, screening employs a relatively simple self-administered questionnaire, whereas, in the second stage, screen-positive participants are subjected to more thorough face-to-face or telephone interviews. This method is less resource-consuming than interviewing all respondents personally, while retaining the diagnostic precision of the personal interview. However, it is probably more at risk of false-negative screening, and sensitivity is expected to be lower, particularly for minor headache complaints, than when all respondents are interviewed personally.

The screening question(s) should be constant throughout the study, and set out *verbatim*. It sets boundaries to the definition of headache caseness; therefore, the study results depend to a large degree on its exact phrasing, and the wording of it is determined by the purpose(s) of the study. A neutral question is “Have you had headache during the last year?”; such a question will include, as cases, those for whom headache is only a minor nuisance, and will therefore yield a higher prevalence than a non-neutral question specifying degree, frequency, intensity or circumstances of its occurrence [12]. Examples of the latter are: “Have you *suffered* from headache?”; “Have you had *frequent* headache?”; “Have you had *bad* headache?”; “Have you had headache *not due to hangover, head injury, flu or common cold*?”

One or more additional questions function as a sieve, separating out participants according to thresholds of frequency (*eg*, ≥1/month), intensity or functional impairment. Through the combination of these questions, a case definition can be arrived at that is more interesting from a medical or public-health perspective. At the same time, it is known how many participants have any headache and what proportions are sieved by each sieve question.

#### Diagnosing headache types

In most studies, whether of headache prevalence or burden, it is of interest to distinguish between different headache types. Diagnostic questions should relate to the criteria of the International Classification of Headache Disorders (the most recent being the 3rd edition, published in a beta version [ICHD-3 beta] but for immediate use [30,31]) because these, by almost universal acceptance, are the common language of definition, and description, of headache disorders. Therefore, unless it is the express purpose of the study to investigate a new or proposed revision of the ICHD criteria, no alternative can be considered.

But there are problems with this. For epidemiological purposes, these criteria must usually be built into a structured questionnaire (see pages 16 and 17), although this is not how diagnoses are usually made in clinic. ICHD criteria were not designed for epidemiological enquiry, and are not particularly well-suited to it. Several deliberations are necessary when planning to employ them in such studies.

First, their strict application requires that all participants are personally interviewed, and in many cases examined, by a competent clinician. In most cases, even if possible, this would be a questionable use of resources. Second, ICHD criteria are expressed in technical language, and must be translated for lay participants without loss or distortion of meaning. Third, and partly consequent upon this, certain criteria distinguishing between migraine and TTH have been noted empirically to pose particular problems in epidemiological surveys. It has been found difficult to gather correct responses on headache duration [2], requiring patients to consider *untreated* attacks, which they may never have, or last had long ago. There are no easy lay explanations of photo- and phonophobia, which are technical concepts. Even more difficult is to specify what degrees of photo- and phonophobia fulfil migraine criteria in ICHD. Headache specialists are trained to sort these things out, but other methods of data collection struggle to do so.

For these three problems there is no perfect solution. Pragmatically, there have to be modifications of these criteria, and, because these involve departures from accepted criteria, they should then be tested and shown to be methodologically sound in a validation study that compares diagnoses obtained by the study instrument with those of one or more headache experts (“gold standard”: see page 20).

A fourth deliberation concerns headache occurring on ≥15 days/month: it is generally recognized that precise diagnosis is difficult at a single encounter with participants (or, indeed, patients in clinic) who have such headache. But they, and especially the proportion of them with MOH, are of particular public-health interest.

A fifth deliberation relates specifically to migraine with aura, because aura is very difficult to diagnose with anything close to certainty by questionnaire. As noted earlier, migraine with aura includes, within ICHD-3 beta, the subforms *migraine with typical aura*, *migraine with brainstem aura* and *typical aura without headache*. Unless migraine aura is a main object of the study, it is unwise to allow the diagnosis of aura to dictate the diagnosis of migraine with aura.

Sixth, and bearing on the seventh, certain questions cannot remain unanswered if a diagnosis is to be made. When it must be established whether a diagnostic characteristic (usually a symptom) is present or absent, the relevant question in a structured questionnaire usually offers only the response options “yes” or “no”, since “don’t know” is unhelpful. But forcing a response from a participant who is uncertain is self-defeating. It is arguable that “don’t know” implies absence, since presence of a symptom creates awareness of it (good examples being photophobia and phonophobia), and only absence leads to uncertainty. This requires empirical testing.

The seventh deliberation relates to “probable” diagnoses defined first by ICHD-II [11] (see below).

#### “Definite” and “probable” diagnoses

ICHD-3 beta, as did ICHD-II before it, allows the diagnosis of “probable” primary headache disorders according to a general rule: when all but one diagnostic criterion for disorder X are met, the diagnosis is “probable X”, *provided that* not all criteria are met for another disorder Y (in which case the correct diagnosis would be Y).

The concept of “probable X” has an important purpose in clinical management, providing a basis for a treatment plan pending later diagnostic confirmation. In epidemiological surveys, later confirmation is not expected: initial diagnoses of probable X have no opportunity to be amended either to X or to another diagnosis. In the specific cases of migraine and TTH, diagnosis of the former depends upon the presence of specific features (*eg*, nausea, vomiting, photophobia and phonophobia, aggravation by physical activity), while diagnosis of TTH depends essentially upon the absence of these same features. By ICHD-3 beta rules, the presence of all but one feature of migraine is not consistent with a diagnosis of TTH, and must lead to a diagnosis of probable migraine. The same is true of probable TTH. Yet there are unavoidable uncertainties in questionnaire-based diagnoses, with the empirical consequence that about half of such diagnoses are “probable” according to ICHD rules [14,15] while, in validation studies conducted in sub-groups of the same populations, fewer than 10% of expert diagnoses are probable.

It is difficult to see that the concept of “probable X” serves any purpose in studies of population health. Rather, its application detracts from diagnostic accuracy [32], and it can be seen why this is so. All questionnaire-based diagnoses of primary headaches are “probable”, partly because of these uncertainties and partly, and in particular, because adequate enquiry to exclude secondary headaches cannot be undertaken. Any distinction in epidemiological studies between “definite X” and “probable X” is arbitrary. Almost all cases of “probable migraine” are of episodic headache that does not meet criteria for TTH, and are therefore more probably migraine than any other type of headache. Likewise, almost all cases of “probable TTH”, not meeting the criteria for migraine, are more probably TTH than anything else. Furthermore, however the distinctions between “definite” and “probable” are made, “probable migraine” and “probable TTH” are *not* separate diagnostic entities, and the practice of considering them as though they were is both illogical and unhelpful; it needs to be strongly discouraged.

As an approach that is both pragmatic and practical in epidemiological studies, we recommend that “definite migraine” and “probable migraine” be reported separately, then combined (“all migraine”), and similarly “definite TTH” and “probable TTH” (“all TTH”). The important proviso is that the diagnostic algorithm (see page 19) applies criteria for these diagnoses in the correct order, which is dictated by ICHD [30].

An important function of validation studies (see page 20) is to provide proof for this approach. Agreement between questionnaire-based and expert (“gold-standard”) diagnoses is usually low for probable diagnoses (empirically, as noted above, questionnaire rates of probable diagnoses are much higher than expert rates), but much higher for definite and probable combined.

In the specific case of MOH, this is diagnosable both in the main survey and in any validation exercise only as an association of medication overuse with frequent headache. Without evidence of causation, all cases are probable MOH.

#### How many different diagnoses in the study?

ICHD-II and ICHD-3 beta describe some 200 different headache diagnoses, including subtypes and subforms. While the principles presented in these recommendations are relevant for all headache diagnoses, in epidemiological studies it is neither feasible nor generally desirable to attempt diagnosis of more than a few of these. From the point of view of headache burden, migraine, TTH and MOH are of the greatest interest.

Multiple diagnoses call for more extended enquiry, which, probably, leads to lower diagnostic precision [27]. Many studies have focused on a single diagnosis (usually migraine), meeting their purpose.

#### Diagnostic algorithm for headache

A diagnostic algorithm, applied later to the recorded responses, separates the interviewer from the diagnostic process. This is usually necessary because the interviewers are not headache experts, and not able themselves to apply diagnostic reasoning. When there are multiple interviewers, an algorithm ensures uniformity in the process.

The algorithmic flow is important. Strictly applied, the criteria for ICHD diagnoses may be mutually exclusive, but this exclusivity tends to be lost with “probable” diagnoses and, especially, with modifications necessarily made to these criteria (see page 19). ICHD actually recognizes that a single headache type can meet more than one set of criteria, and counters this possibility by instructing that diagnoses should be made in a hierarchical sequence [11,30]. Secondary headaches, including MOH, should be diagnosed before primary headaches, and migraine should be diagnosed before TTH. A diagnosis of definite migraine is given when all criteria are met for migraine and, otherwise, the algorithm should accord precedence to TTH over probable migraine and, finally, probable TTH.

A diagnostic algorithm following these principles is attached to the diagnostic module of the HARDSHIP questionnaire (see page 21), which is published alongside these recommendations [29].

#### How many diagnoses in each individual?

Questionnaires can address only one headache disorder at a time, while participants in epidemiological surveys may have multiple headache types and, accordingly, more than one headache diagnosis may be applicable in each individual. Since headache diagnosis is based on history and recall, there is potential for confusion if a participant has more than one headache type in mind, and this needs to be eliminated as far as possible. Furthermore, it may be difficult for the participant with several headache types to remember which features belong to which headache type, and attribute them accordingly. For the interviewer, it is a great challenge to sort this out correctly. Headache specialists are trained to do this in the clinic, but otherwise, and certainly in surveys, it is problematic. If only one diagnosis is to be made when more than one headache disorder is present, which should it be? A solution, valid for public-health purposes, is to ask the participant to identify in his or her mind the most bothersome headache (*ie*, the one that the participant believes interferes most with his or her life), and focus solely on this when responding. This language will tend to favour migraine, which is likely to be the most bothersome headache when multiple types are present (see discussion in the next section).

This approach is adopted in the HARDSHIP questionnaire (see page 21) [29].

#### Relevance of diagnosing different headache types

This approach carries a small penalty. Prevalence studies that focus on the most bothersome headache will under-count headache types that tend to be less bothersome: for example, almost all participants who have both migraine and TTH will focus on the former, so that TTH is systematically neglected. This should be acknowledged. There is an argument for extrapolating the reported prevalence of TTH in those without migraine to those with.

Of course, a questionnaire can be applied a second (and third) time to include all headaches that participants can separately identify. This has been done [33,34], but most participants find it somewhat trying. When it is done, it should be reported how the multiple diagnoses in single individuals overlap (*ie*, how many participants have migraine only, how many TTH only, how many migraine and TTH).

Making multiple diagnoses markedly increases the number of questions that must be asked. This tends to discourage participation, and leads to a lower participation rate. All studies should carefully assess the value of making specific headache diagnoses. For example, and in particular, what is gained in a study to support needs-assessment by distinguishing between migraine with and without aura? For the purpose of assessing population headache burden, to distinguish at all between different headache types may be irrelevant. In fact, burden estimation in individuals with multiple headache types cannot, even when diagnoses are correct, realistically attribute burden in parts to each type. For public-health purposes, burden can be attributed to “headache” in general [35]. If, instead, it is attributed to the most bothersome headache reported by each individual, it is unlikely that the resultant over-estimation as far as that headache type is concerned will be substantial.

#### Validation of the questionnaire

The key objective of validation is to prove the diagnostic capability of the diagnostic questionnaire, and is necessary whenever diagnostic accuracy is important and diagnoses are not made by headache experts in face-to-face interviews. The importance of validation is increased because questionnaire diagnosis requires some modifications of ICHD diagnostic criteria (see page 19).

Diagnostic validation is performed in a separate sample selected by identical methodology to the main sample, or in a randomly or consecutively selected sub-sample of participants in the main study. In the former case, validity can be confirmed before resources are committed to the main study; otherwise, the latter method conserves resources by not requiring additional participants. In either, stratified sampling according to caseness and diagnosis (no headache, migraine, TTH or MOH) may be employed to ensure adequate numbers with each diagnosis and without headache. Participants in the validation sample or sub-sample are re-interviewed and diagnosed by a headache expert applying his or her clinical skills, while being ignorant of the questionnaire diagnoses (a headache expert in this context is a doctor who has clinical experience of diagnosing headache according to the ICHD criteria, and who knows the culture and is fluent in the native language(s) of the population of interest). Re-interviewing should be done soon (no more than 1 month) after the original diagnosis, so that any discrepancies are unlikely to be due to a change in the headache condition itself.

Questionnaire-derived diagnoses in headache cases are compared with those of the expert(s), the latter being considered the “gold standard”. This allows calculation of sensitivity, specificity, positive and negative predictive values and kappa value of the diagnostic instrument for each diagnosis [36]. The precision (*ie*, the width of the confidence interval) of the estimate for a certain diagnosis is dependent both on the total number of persons included in the validation study and on the number of persons with the diagnosis. Validation studies performed in the last decade typically include from 180 to 500 persons drawn randomly from the main study, and have given relatively narrow confidence intervals for all the most common headache diagnoses [14,15,36-38] except, perhaps, MOH [36]. Validation of the screening question (“Have you had headache …?”) is achieved in those who answered “No” to it.

Validations preferably employ face-to-face interviews by the headache expert, but telephone interviews lose little and are an acceptable resource-conserving alternative when respondents are widely spread geographically.

Validation *cannot* be performed among headache patients in clinic, who are not representative of the population of interest in terms of their headache disorders and, probably, in a range of other relevant factors. They commonly have more knowledge of headache, and perform differently from non-clinic populations when answering questionnaires because they have rehearsed their histories.

As noted previously, validation is not always possible [3]. In countries where there are no headache experts, there is no gold standard available. What should be avoided in such circumstances is the invention of a new and untested questionnaire; instead, a diagnostic questionnaire that has been used and validated in multiple languages and cultures can be assumed to have some validity in countries, in languages (subject to good translation) and among cultures where direct validation cannot be performed. The alternative is that research, which is of public-health importance, can never be commenced.

A validation study can inform and optimize the diagnostic algorithm: in other words, small amendments can be made to the algorithm and the comparison remade between questionnaire-derived and expert diagnoses, which may or may not lead to improvement. An example might be to exclude from the diagnostic criteria for migraine the minimum duration of 4 hours, which has been found empirically to cause problems [2].

What is optimal depends on the purpose of the study. There is a trade-off between sensitivity and specificity, in the sense that as one increases the other decreases. In genetic studies, for example, where diagnostic certainty is most important, the algorithm that gives the highest specificity may be preferred. For most purposes, the algorithm that gives the highest overall diagnostic accuracy (sum of sensitivity and specificity) is preferable.

#### The HARDSHIP questionnaire

The diagnostic module of this survey instrument, published as an example alongside these recommendations [29], follows the principles set out above. Based originally on ICHD-II and now on ICHD-3 beta, with necessary modifications (see page 19), it has been validated against headache experts’ diagnoses in multiple and different settings.

HARDSHIP first enquires into the presence or absence of headache, ever and in the previous year (screening questions). When headache is reported, the questionnaire recognizes the diagnostic difficulty when headache occurs on ≥15 days/month (see page 18), and that making more than one diagnosis in each individual is problematic. It assumes that, from a public-health point of view, the important diagnoses are migraine and TTH, but also acknowledges that a very large burden is carried by people with headache on ≥15 days/month. Hence, it specifically identifies these people, and asks them about possible medication overuse (which of itself is of public-health importance). In all others, headache is assumed to be episodic. The next question asks whether participants believe they have only one type of headache or more than one; when there is more than one, subsequent questions are directed to the most bothersome type (see page 20), and it is made explicit that, because only one headache type is reported by each participant, diagnoses are mutually exclusive.

### Pilot study

#### Principles

A pilot study:

• Identifies problems before full commitment of resources, and therefore:

•Supports optimum study design, logistic planning and effective conduct of the main study;

•Avoids potentially major wastage of resources;

•Is ethically desirable;

• Helps in training of research staff, especially interviewers.

### Commentary

It is both highly sensible and ethically desirable to perform a pilot study in order to detect and forestall problems in the main study. Unlike a pre-pilot study (see page 21), a pilot study cannot use a patient sample. It may, for logistic reasons, employ an element of convenience sampling (*eg*, being restricted to nearby geographical areas), but otherwise it uses all of the proposed methods of sampling, access, engagement and data collection. It tests practicalities, comprehensibility of the instrument and overall feasibility of the study. With regard to the instrument, it should identify which items (questions or responses) interviewers may need to clarify, and the extent and nature of clarifications to be allowed. With regard to feasibility, it should provide estimates of the expected non-contact and non-participation rates.

## Burden estimation

### Principles

Enquiries into burden should be:

• Relevant;

• Comprehensive (in relation to the purpose of the study);

• Comprehensible to the respondent;

• Amenable to analysis.

### Commentary

#### Relevance and comprehensiveness

“Relevance” implies that measured burden must be attributable to headache, and not to any other cause (including comorbidity). Otherwise the enquiry is spurious and findings are invalid. For this reason, inclusion of instruments to register important comorbid conditions is warranted (*eg*, psychiatric comorbidity).

“Comprehensiveness” requires that *all* relevant components are measured in order to give a full account. However, the purpose of the study can legitimately restrict the enquiry to specific elements (for example, financial burden). When this is so, explanation is necessary.

All or any of the components of burden described earlier (see page 3) may be relevant and appropriate objects of enquiry in burden-of-headache studies.

#### Comprehensibility and analysis

Most but not all of the elements of burden are amenable to enquiry by well-designed questionnaire. The HARDSHIP questionnaire [29] (see page 21), for example, incorporates enquiry into most of them in a modular design, which means the different elements can be included or not. It has been tested for comprehensibility and successfully employed in different cultures and languages in Russia, China, India, Pakistan and ten countries of the European Union [14-17,39] and in Saudi Arabia, Zambia and Ethiopia (not yet published). In the commentary below, references are made to specific questions in HARDSHIP.

Some elements of burden are not quantifiable, but amenable only to qualitative (descriptive) analysis.

#### Symptom burden

See HARDSHIP questions 14, 15, 20, 21/23, 24, 29–32, 36 and 37.

Pain burden can be quantified at individual level as a product of intensity, frequency and duration, and at population level as the product of the average among individuals and prevalence. But it is subjective, quantity estimates are inexact and there are no meaningful units in which it may be expressed. Other symptoms such as nausea, photophobia and phonophobia are almost impossible to quantify at individual let alone population level, but occurrence of each can be recorded and expressed as a frequency.

#### Disability burden

See HARDSHIP questions 38–44 and 58–62.

There is correlation between symptoms and disability, which allows the latter to be measured in place of the former. Arguably, disability and lost productivity occur in addition to symptoms, so measurement of disability instead of symptoms underestimates their impact. Less arguable is that disability occurs only as a consequence of symptoms, and to measure both is a form of double-counting.

Disability attributed to headache is also difficult to quantify completely. Common proxies are lost time and reduced productivity, for which well-validated instruments exist [40,41]. Both can be expressed in meaningful units (days/year), but both are problematic. Lost time (absenteeism from paid work, for example) reflects behavioural response to impairment rather than disability: except in extreme cases, absenteeism is a choice. Reduced productivity is rarely exactly measurable, and at best can be approximately estimated by the person affected. A better measure conceptually may be the disability-adjusted life year (DALY), or the disability component of it, the YLD (year of life lost to disability). Attribution of YLDs to headache depends, essentially, on prevalence estimation and disability weighting. Since disability weights (DWs) are diagnosis-specific, the process is underpinned by diagnostic accuracy. Revised DWs have been developed as part of the Global Burden of Disease Study 2010 [1].

#### Individual financial burden

To the extent that financial cost arises from lost wages, as a consequence of absenteeism, it should be directly measurable and easily expressed, and is a relevant and important component of the burden of headache for many people. However, housewives and unemployed people may have no income to lose. In other cases, this cost is only a small part of the personal burden because it is largely borne by employers or insurers, contributing instead to the very high *societal economic burden* (see page 23).

A personal cost burden may also arise from health-care resource consumption (see page 23).

#### Interictal burden

See HARDSHIP questions 64–66, and 90–101.

The importance of interictal burden lies in the fact that it is continuous, rather than present only during attacks occurring perhaps every 30 days. This means both that it should not be ignored and that, if over-estimated, it will distort overall burden quantification.

Interictal burden impairs wellbeing and, potentially, quality of life. It is perhaps adequately, if not specifically, captured by measures of these.

#### Cumulative burden

See HARDSHIP questions 51–57.

It would appear that components of burden that accrue, cumulatively, over a lifetime cannot be fully assessed until late in lifetime. Furthermore, attribution may be difficult and uncertain. Nevertheless they are of obvious importance in a full account of burden of headache, and can be addressed in questionnaires.

#### Overall individual burden

See HARDSHIP questions 67–74.

An overall summary measure of burden is unlikely to be comprehensive, but the concept is attractive for its simplicity. It may also be useful for specific purposes.

One such measure is willingness-to-pay (WTP). The enquiry is along the lines of: “Imagine that there is a treatment you can buy. If you take it, your headaches will no longer bother you. How much would you be willing to pay (per week or month) for this treatment?” Responses are usually adduced through the “bidding game” method. This begins by first asking whether the respondent would pay a modest amount (set in context) for the treatment. If the answer is “yes”, the interviewer increments the bid until the answer is “no”; the last sum of money receiving a “yes” response is the WTP result. If the initial answer is “no”, the interviewer reduces the bid in steps until the respondent says “yes”; the first sum of money receiving a “yes” response is then the WTP result.

This form of enquiry has been used to assess sustainability of health-care initiatives in resource-poor countries [42]. It has wider potential applicability, since it puts a value, in monetary terms, to subjectively perceived burden in all its elements. Use for this latter purpose requires validation, especially because willingness-to-pay is clearly not independent of ability-to-pay. In addition, there may be a disconnection between what people say they will pay and what they actually will pay when confronted by the prospect in reality.

#### Burden on others, including carer burden

See HARDSHIP questions 75–86.

Again, this can be addressed in questionnaires, although subjective interpretations are unavoidable. A full account may necessitate enquiries among the others, which in practical terms may be possible only among close family members.

Lost work time of carers is potentially important.

#### Health-care resource consumption

See HARDSHIP questions 45–50.

It is relatively easy to enquire into health-care usage, and to establish who pays for it (the patient, insurers or society via the State). It is not so easy to attach accurate costs to individual items of health care when these costs are not borne directly, out of pocket, by the patient. This needs to be done, however, and may necessitate separate research into health-care costs in the country or region in question [6].

#### Societal economic burden

See HARDSHIP questions 58 and 59.

By far the greater part of the financial cost of headache is the indirect cost of absenteeism and reduced effectiveness at work. This cost may be borne by individuals (see above); but commonly it falls largely, if not entirely, upon employers and/or insurers and is a cost to national economies and becomes a part of the societal economic burden. If disability measures use absenteeism and reduced effectiveness at work as proxies (see above), reasonable estimates of this cost can be made with knowledge of average wage-rates (age- and gender-corrected) for the population in question.

More is said about societal economic burden in the next section.

### Studies focused on economic burden of headache

#### Principles

Comprehensive enquiries into economic burden:

• Should include both direct and indirect costs;

• Must have reliable data sources for attributing costs;

• Must be based on population studies, not patient samples;

• Should preferably be planned and performed in collaboration with a health economist.

### Commentary

There are references above to financial costs of headache, which may fall either on individuals or directly on society. A full account of financial cost includes both. It also includes both direct costs (what the patient or someone else has to pay for) and indirect costs (losses of income or productivity) (see below). Describing the burden of headache on society in purely economic terms has two main purposes:

a) It allows evaluation of headache disorders in comparison with other diseases of public-health importance laying claim to health-care resources;

b) It may show how much can be gained by improving health-care services for headache patients.

#### Direct costs

Direct costs include the costs of medicines and other treatments, of consultations (including transportation costs when these are necessarily incurred), of diagnostic investigations and of hospitalizations. They may be covered by the patient, by the State, by a commercial health-insurance system or in parts by any two or all three.

#### Indirect costs

Indirect costs are dominated by loss of productive work time, due either to absence from work or to reduced efficiency when working with headache. This cost may be borne by individuals (see page 23), but in many societies it falls mostly upon employers and/or State or commercial insurers.

Other types of indirect cost are hard to capture, and therefore rarely described: among these are lost career opportunities because headache has interfered with school and work (a type of cumulative burden [see page 23]) and lost productivity among partners who become carers.

#### Measuring economic burden

See HARDSHIP questions 17, 18, 39–50, 58–62 and 80.

If appropriate items of economic relevance are included in the questionnaire, it is possible to estimate the cost of headache by the “bottom-up” approach: *ie*, cost is calculated for each person with headache in the sample, and all costs are summed to give the total cost within the sample. This can be extrapolated to the whole population of interest.

The bottom-up method requires a price tag to be placed on each item (*eg*, on each dose of different medicines, on each consultation, on each diagnostic investigation, on a day and/or night in a hospital bed, on a lost work-day, *etc.*). These prices must be obtained from reliable sources. The method is generally viewed as more inclusive and accurate than the alternative top-down approach, by which the overall cost is calculated from publicly available statistics (*eg*, total sales of migraine medicines, summary statistics on health-care utilization and records of sick-leave due to headache).

Ideally, bottom-up cost estimates are made through population-based studies in which both prevalence of headache and associated costs are determined simultaneously. Costs for this purpose cannot be assessed in patients (users of health-care resources) because they are not a representative group; they usually have more severe or complex headache disorders, so that extrapolation to a broader population of interest will give a spuriously high total cost [6], and they may be self-selecting (biased) in other ways.

It is recommended to involve a skilled health economist in the design, planning and analysis of a study focusing on economic burden.

### Timeframe of burden estimation

As observed earlier, the limitations of *recall* are an important factor in the generation of information bias. They are greater in the elderly, so introducing differential bias (see page 9).

Recall of headache occurrences and, more so, recall of the characteristics and consequences of each occurrence are presumably more accurate over the previous 3 months than over the last year. For this reason, burden questions have commonly been limited to a 3-month timeframe, as in the Migraine Disability Assessment (MIDAS) [40] and Headache-Attributed Lost Time (HALT) [41] questionnaires. The adoption of 3 months is a compromise, constrained on one side by the limits of recall and on the other by the purpose of the enquiry. Both instruments have in mind the assessment of an individual patient for therapeutic reasons and, to establish that person’s burden, must cover a period long enough to be representative. In *large-group* studies, quite different considerations apply: since 3 months may be too long for reasonably accurate recall of productivity losses, shorter periods of one month or even one week should be considered.

Very short and recent timeframes, such as headache yesterday, avoid recall problems almost altogether. While an estimate of 1-day prevalence does not describe the proportion of the population with an active headache disorder, enquiries focused on headache yesterday can yield very accurate information on burden. If the sample is large enough (1-day prevalence of an episodic headache disorder being obviously much lower than 1-year prevalence), they offer a rather precise estimate of the level of headache suffering in the population on a particular day, and therefore on any day. Hence headache yesterday can very accurately describe the population headache burden.

## Data collection and storage

### Principles

Data management and storage processes should:

• Be designed to minimize error;

• Be carried out meticulously;

• Respect the privacy of participants and accord with the laws and regulations for data storage in the country.

### Commentary

Huge effort and resource-expenditure go into error-free data collection; errors should not be introduced subsequently by less-than-meticulous data management.

However collected, data should be recorded in the most practical way: usually initially on paper, but sometimes directly into computer. In the former case, data must later be transferred from the source documents to computer and, in this process, rigorous quality controls are essential. Ideally, all data are entered twice, by two independent persons (double data entry), producing two datasets that are compared to detect errors, each of which is resolved by reference to the relevant source document. As a resource-conserving minimum, a subset of the data (*eg*, 10% of items) is entered twice and, by comparison of the two data subsets, an error rate is estimated. Taking due account of the uncertainty surrounding this estimate, a decision can then be made as to whether this error rate is acceptable (*ie*, will have negligible influence on the results to be reported); if not, full double data entry is necessary.

As always, personal data should be handled and stored safely, respecting the privacy of participants and in accordance with the laws and regulations for data storage in the country (see Ethical Issues, page 4).

## Special issues

### Studies of special groups

Some studies concern not the general population but, instead, particular subgroups of it (*eg*, children and/or adolescents, the elderly). These may introduce additional issues for consideration.

### Children and adolescents

In countries with obligatory schooling, representative samples of children of school age can be obtained by selecting all, or a random sample, of the pupils of a representative sample of schools (a cluster-sampling method). These schools should reflect the variation of the country or region with regard to socioeconomic status and area of habitation (rural or urban). Access is greatly facilitated, since the pupils can be contacted while at school. Engagement, and interviews, may be undertaken by specially trained interviewers, or by a teacher or school nurse. For small children, some information will have to be given by parents or teachers.

For studies on children or youths below the age of majority, consent to participation must be obtained beforehand from parents or guardians. This requirement cannot be bypassed; but, if consent is sought by letter from or through the school – the usual method – there may be a major diminution of participation-rate through non-response. The apparent advantages of sampling via schools, rather than by door-to-door visits in the community, may be outweighed by this factor.

Somewhat different diagnostic criteria for migraine apply in children (although still based on ICHD). Diagnostic instruments require modification accordingly, and these and other measuring instruments must reflect the language and comprehension skills of the age group. Validation of the diagnostic method is as important as in studies on adults, and the headache expert in these studies should have experience in diagnosing headache in children.

### The elderly

Studies in elderly populations (usually defined as >65 years of age) should take note of the following, all to be expected:

• Lower prevalences of primary headache disorders;

• Higher prevalences of secondary headaches;

• More comorbidities;

• Reduced mental capacity (potentially affecting engagement and data collection);

• Reduced physical capacity and less participation in the workforce (potentially affecting the way in which headache has impact, and, therefore, estimates of burden).

For these reasons, more factors come into play when attempting to draw a representative sample in this age group, and the sample size may need to be enlarged. The enquiry may need to focus more on secondary headaches, at least to avoid their misdiagnosis as primary headache disorders when the latter are the object of interest. It may also need to give greater consideration to comorbidities, which may be confounders in estimations of burden. Adjustments for comorbidities will be more important in the analysis stage.

### Studies of rare headaches

The prevalence of rare headaches is difficult to assess because a large sample size is needed (see page 11). Also, because of limited experience with such headaches, they may be difficult to identify through self-administered questionnaires; a headache expert must usually make the diagnosis. Such a study has been performed for cluster headache [43].

Rare headaches are unlikely to be of public-health interest unless severe, which opens up a possible alternative method for case-ascertainment: the screening of large clinical populations (*eg*, general practitioners’ clinical files) for potential cases, and then a more thorough clinical expert interview of these for diagnostic confirmation [44]. This may work well for severe headache disorders such as cluster headache or trigeminal neuralgia that *demand* medical attention, in societies with good access to primary care and well-kept electronic (*ie*, searchable) records. Diagnoses (and even symptoms) may not be correctly recorded but, nonetheless, case-ascertainment through careful searching can be virtually complete. The denominator in such cases is the general population.

Most published prevalence estimates of rare headaches are of lifetime prevalence, because cases are more numerous with this definition of caseness (see page 18); but current prevalence (1-year or 1-day [see page 4]) is likely to be of greater interest.

### Stand-alone studies versus large health surveys

These recommendations call for a relatively long questionnaire, suited to stand-alone studies that are dedicated wholly to assessing headache prevalence and burden. For many scientific purposes it is both feasible and interesting to include headache in large health surveys that aim to investigate multiple disorders and/or other health-related issues.

Large health surveys assess headache in a wider context and may include very large populations because more resources are put into them. They can include comorbidities and associations with potential causes and risk factors, an aspect of enquiry that stand-alone studies are rarely capable of. On the other hand, large health surveys cannot feasibly include a full burden-of-headache questionnaire; instead, the necessary minimum is a valid screening question for active headache disorder, some questions allowing determination of severity (frequency, intensity and duration), and ideally a question set for the diagnosis of migraine and TTH (as mutually exclusive diagnoses). Validation of these questions remains as important as in stand-alone studies.

## Reporting the study

### Principles

• Poor reporting diminishes the value of otherwise good studies.

• Reference to the STROBE statement is recommended.

• Methods should be reported in sufficient detail to permit replication (which may require that they be published separately).

• Results should be reported in accordance with the study’s purpose(s).

• Discussion should show that the study objective was achieved, or explain why it was not.

### Commentary

All too often, the value of otherwise good studies, consuming substantial resources, is diminished because they are poorly reported. This is true both for methods and results. The STROBE statement gives excellent recommendations for how epidemiological studies should be reported [7,8]. Particularly relevant for headache prevalence and burden studies are the parts on cross-sectional studies.

#### The introduction

This should briefly state the scientific question or hypothesis addressed by the study (objective), why this was of interest and the study done, the scientific background (what was known beforehand) and any argument(s) that justified the choice of study design (*eg*, cross-sectional, case–control, cohort).

#### Description of methods

Methods need to be reported in sufficient detail to permit replication of the study. Especially for large studies likely to result in several papers, it may therefore be advisable to write a first and separate methodology paper, so that subsequent papers can refer to it while concentrating on the results. The validation study, and its results, should usually be part of this. When a non-participation study has been performed, the methodology paper should also contain the results of this and discuss their potential impact on the main results.

In all cases, necessary items for inclusion are:

• Ethics approval;

• Study design (*eg*, cross-sectional, case–control, cohort);

• Case definition;

• How the population of interest was defined;

• How the study sample was selected;

• The sampling method, and how participants were identified, accessed and engaged;

• Sample size calculation;

• The number of interviewers, their background, and training;

• The study instrument (to be adequately described), including the exact wording of the screening question;

• Description and results of any validation study;

• The diagnostic algorithm;

• In a separate statistical section, a description of statistical methods and an account of subgroup analyses (each clearly identified as planned before the study or conceived after review of the data, as applicable).

#### Presentation of results

The participation rate (see page 14) is an important result and should always be reported. Further, all reasons for non-participation (see Figure 1) should be given, and a flowchart of participation should account for everyone in the pre-selected sample (Figure 2).

Principal findings need to be set out in tables and not only as graphics (pie or bar charts, *etc.*), because it is often difficult to extract exact values from the latter.

Prevalence estimates *must* specify their timeframe (see page 17). They should be reported for all headache and/or each of the headache types or subtypes of interest. It should be clear whether diagnoses were mutually exclusive or, alternatively, one person could be diagnosed with multiple headache types (or subtypes). In the former case, it should be easy for the reader to add up the types or subtypes to 100% of all headache (see page 20). Estimates should be reported with descriptors of uncertainty (*eg*, 95% confidence intervals), and both unadjusted and adjusted for differences between the age- and gender-compositions of the sample and those of the population of interest, if known. They should further be reported for each gender separately and for both combined, and, in each of these groups, according to age categories (usually each 5 or 10 years). Additional breakdowns (*eg*, urban *vs* rural habitation, educated *vs* uneducated, wealthy *vs* poor) may be of interest. Multivariate analyses will show which of these have true associations (but not that they are risk factors since causation has not been established).

Missing data should be described and enumerated. Always, some participants do not answer all questions and/or some answers are unintelligible or otherwise lost. The amount of missing data can affect generalizability of the results.

Reports of prevalence alone are of limited interest: some measures of burden should be reported in most prevalence studies. For whatever are reported, it is often of interest to differentiate between genders, age groups, urban and rural populations, wealthy and poor, *etc*.

#### The discussion

This should summarize the key results, focusing first on those relevant to the protocol-defined hypotheses and objective, and their analysis, then on secondary hypotheses generated by the data. Methodological limitations and strengths should be evaluated. In particular, it is important to discuss the effect the participation rate, and all reasons for non-participation, may have had on the representativeness of the study sample, taking into consideration the results of the non-participation study, if one was performed. Other potential sources of bias should also be carefully evaluated. This should lead to an assessment of the generalizability of the results, and the influence of all methodological limitations on the conclusions to be drawn.

Finally, the results should be put in the context of what was previously known, identifying the new knowledge that has been acquired and the value and the implications of it for people with headache, health-care providers and/or health-care policy-makers. It is sometimes helpful to suggest new studies that are needed in the light of this new knowledge.

## Evaluating the quality of studies

These principles can be applied not only to the design and conduct of new studies but also to the quality evaluation of published studies.

A scoring instrument has been developed for this purpose (see Table 3). It emphasizes representativeness of the sample with regard to the population of interest, the sampling method, sample size, participation rate, access, validation, use of diagnostic criteria and description of timeframe. Each quality factor has a maximal score of 4, and the maximal total score is 32. For factors crucial to study quality, a heavy penalty of -4 is applied when no information at all is provided: serious flaws in any of these can irredeemably invalidate the results, even when all other aspects of the study have been adequately performed and reported, and the quality assessment must reflect this.

**Table 3:** Criteria for evaluating the quality of headache prevalence studies

**Quality factor**	**Score**				
	**-4**	**1**	**2**	**3**	**4**
**Sampled population**	Not stated, or clinic population, or members of patient organization	Selected population (*eg*, health-plan members, work-place, college students) [increase score by 2 if this met the specific purpose of the study]		General population or community-based sample from defined region within a country, or school-based (for children)	General population or community-based sample from whole country
**Sampling method**	Not stated	No (or failed) attempt to secure representativeness		Random sample uncorrected for population demographics	Total defined population, or random sample corrected for population demographics
**Number of respondents**	Not stated	250-1,000	1,000-1,500	1,500-2,500	>2,500
**Participation rate**	Not stated, or <40%	50-59%	60-69%	70-79%	>80%
**Access**	Not stated	Self-administered (unsupervised) questionnaire	Telephone or face-to-face interview by untrained or unspecified interviewer(s)	Telephone or face-to-face interview by trained lay interviewer(s), medical students or nurses	Face-to-face interview with headache expert [reduce score by 1 if interviews restricted to screen-positive sub-sample]
**Validation of diagnostic instrument**	Instrument not specified or not validated	Validated, but sensitivity and/or specificity <60%	Validated, but sensitivity and/or specificity <70%	Validated only in screen-positive sub-sample, or in clinic or unspecified sample, but sensitivity and specificity ≥70%	Validated in target population or similar, and sensitivity and specificity ≥70%, or all diagnoses made in face-to-face or telephone interviews by headache expert
**Diagnostic criteria, and application of “probable” diagnoses**	Not stated	Stated, other than ICHD	ICHD (or reasonable modification), but uncertain or inappropriate analysis of “probable” diagnoses		ICHD (or reasonable modification) with clear exposition regarding “probable” diagnoses
**Prevalence time frame**		Not specified or not appropriate to the study purpose	Not specified, but terminology implies “present” or “current” or “recent” headache	Other specified period appropriate to the study purpose	Point, 1-day, 1-year or lifetime

Possible range is -27 to +32.

Quality thresholds: <9: unacceptable (data of no value); 9–23: acceptable when no other data are available; 24–27: good; ≥28: very good.

## Conclusions

Population-based burden-of-headache studies contribute essentially to our understanding of disease origins, patterns, aetiology and risk factors, inform needs-assessment and underpin service policy; they must be of high quality. Many factors that influence quality have been noted to be inadequately addressed in past studies [3]. These, and others of potential importance, are made the subject of recommendations accompanied by detailed explanatory commentary. The expertise contributing to this document was extensive, both theoretical and practical, and drawn from all regions of the world; while it is not a textbook on epidemiology (general principles are dealt with elsewhere), the recommendations should provide helpful and authoritative guidance for most purposes related to headache.

## Abbreviations

CATI: Computer-assisted telephone interview; DALY: Disability-adjusted life year; DW: Disability weight; ERB: Ethics review board; HALT: Headache-attributed lost time; HARDSHIP: Headache-attributed restriction disability, social handicap and impaired participation; ICHD: International classification of headache disorders; LTB: *Lifting The Burden*; MIDAS: Migraine disability assessment; MOH: Medication-overuse headache; SD: Standard deviation; STROBE: Strengthening the reporting of observational studies in epidemiology; TTH: Tension-type headache; WHO: World Health Organization; WTP: Willingness to pay; YLD: Year of life lost to disability.

## Competing interests

LJS, GLB, RJ, ZK and TJS are directors and trustees of *Lifting The Burden*. There are no other conflicts of interest.

## Authors’ contributions

TJS and LJS conceived the idea and assembled the group of experts. LJS and TJS reviewed the literature. All authors were members of the expert consensus group and contributed to the formulation of these principles and recommendations. TJS and LJS drafted the manuscript. All authors reviewed the manuscript in various drafts and approved the final version.
